# Applications of machine learning in glaucoma diagnosis based on tabular data: a systematic review

**DOI:** 10.1186/s42490-025-00095-3

**Published:** 2025-08-01

**Authors:** Mohammad Hasan Shahriari, Farkhondeh Asadi, Hamid Moghaddasi, Arash Roshanpour, Farideh Sharifipour, Zahra Khorrami

**Affiliations:** 1 Department of Health Information Technology and Management, School of Allied Medical Sciences, Shahid Beheshti University of Medical Sciences, Tehran, Iran; 2 Ophthalmic Epidemiology Research Center, Research Institute for Ophthalmology and Vision Science, Shahid Beheshti University of Medical Sciences, Tehran, Iran; 3 Department of Computer, Yadegar-e-Imam Khomeini (RAH), Shahre Rey Branch, Islamic Azad University, Tehran, Iran

**Keywords:** Glaucoma, Decision support system, Artificial intelligence, Machine learning, Deep learning

## Abstract

Glaucoma is a leading cause of irreversible blindness, necessitating early and accurate diagnosis to prevent vision loss. Traditional diagnostic methods often suffer from subjectivity and variability, emphasizing the need for more reliable approaches. This study evaluates the application of machine learning (ML) techniques in glaucoma diagnosis, analyzing their effectiveness and identifying the most promising methods and datasets. A systematic review of five major databases was conducted, selecting 35 studies based on predefined criteria. The findings reveal that structured data, including optical coherence tomography (OCT), visual field (VF) tests, and demographic factors, significantly enhance diagnostic accuracy. ML models such as support vector machine (SVM), deep learning (DL), random forest, and ensemble methods demonstrated accuracy ranging from 76 to 98.3%, with AUC values between 52.5% and 99%. Despite these advancements, challenges such as data imbalance and limited sample sizes impact model generalizability. The results highlight the potential of ML to improve glaucoma detection, though further research is needed to enhance data quality and model validation for broader clinical applicability.

**Supplementary Information:**

The online version contains supplementary material available at 10.1186/s42490-025-00095-3.

## Introduction

 Glaucoma consists of progressive eye disorders characterized by the degeneration of retinal ganglion cells and retinal nerve fiber layers (RNFL), ultimately leading to irreversible optic nerve damage and vision loss. It is a significant public health concern, being the second leading cause of blindness worldwide [ 1, 2 ]. As of 2020, around 80 million people globally were affected by glaucoma [ 3 ] and this number is projected to reach 112 million by 2040 [ 1 ]. The American Glaucoma Society reports that 2.7 million Americans have glaucoma, yet only half of them are aware of their condition [ 4 ]. The annual costs for glaucoma treatment and healthcare in the U.S. is approximately $2.5 billion [ 5 ]. Early diagnosis is crucial in reducing the risk of permanent vision loss and mitigating the disease’s impact on the healthcare system [ 6 ].

Evidence-based guidelines for diagnosing glaucoma advocate a multifactorial approach that involves regular ophthalmologist eye exams and personalized diagnostic tests based on patient characteristics and history [7–9]. This includes evaluating clinical findings, imaging to assess functional and structural changes, and considering risk factors like age, increased intra ocular pressure (IOP), myopia, and family history [3, 10, 11]. Glaucoma is a multifactorial disease whose diagnostic process is complex, subjective, and resource-dependent [2, 12, 13]. There is an overlap in the examinations and tests of glaucomatous and non-glaucomatous patients. Some individuals may develop glaucoma with IOP levels within the normal statistical range, known as normal-tension glaucoma. In these cases, relying solely on IOP may lead to missed diagnoses [3, 14]. Given the complexity and multitude of factors involved in diagnosing glaucoma, various data sources are integrated to provide a comprehensive assessment [15].

Considering the necessity of analyzing multiple factors, clinical decision support systems (CDSS) can help address this challenge [16, 17]. Artificial intelligence (AI) and machine learning (ML)-based CDSS can learn from large volumes of data to identify patterns and trends that are difficult or impossible for humans to detect [18–20]. Recently, AI and ML methods have been successfully used in clinical data analysis and image processing [21, 22]. In the field of ophthalmology, several AI-based approaches have been proposed for diagnosing diseases such as diabetic retinopathy, diabetic macular edema, and keratoconus [23–25]. Utilizing the capabilities of ML algorithms presents promising solutions for improving the accuracy of early glaucoma diagnosis.

Research on ML-based CDSS for glaucoma diagnosis has been ongoing for about three decades [26–28]. Some studies have reported automatic glaucoma diagnosis using fundus and optical coherence tomography (OCT) images [29–34]. Other studies have developed AI models for glaucoma diagnosis based on structured tabular data in electronic health records (EHR). In a large-scale study utilizing EHR’s from 650 U.S. healthcare facilities, Raju et al. implemented a predictive analytics framework for early glaucoma diagnosis (AUC = 0.80) [35]. Lee and colleagues developed sophisticated models integrating demographic factors and OCT data, achieving high diagnostic accuracy (AUC = 0.93) [36]. Also, Oh et al. further advanced this field by introducing an interpretable ML model that not only achieved superior diagnostic performance (AUC = 0.94) but also incorporated an explanation system to elucidate individual predictions, enhancing clinical transparency [37].

Given the importance of early diagnosis of glaucoma in preventing blindness, the multifactorial nature of this disease, and the application of ML methods in this field, the research team decided to conduct this review. The focus is on studies that are based on structured tablular data. Additionally, it aims to discuss the potential impact of ML on improving the accuracy of glaucoma diagnosis, patient care, and the evolving landscape of glaucoma research.

## Methods

This systematic review was conducted to identify and analyze studies related to glaucoma diagnosis using ML methods and structured data.

### Literature search

The present systematic review was conducted by searching five databases: PubMed, Scopus, Web of Science, ScienceDirect, and Institute of Electrical and Electronics Engineers (IEEE). The search utilized the following keywords: “Glaucoma”, “Artificial intelligence”, “Machine learning”, “Deep learning”, “Decision support”, “Decision aid”, “Computer-assisted”. Keywords were selected using PubMed Mesh and related studies. The search queries are presented in Table 1. The search was performed on 28th Dec 2024, and was limited to articles published from from 2010 to 2024.

### Eligibility criteria

This review included studies that met the following inclusion criteria:

1. Original research articles focused on the diagnosis or classification of glaucoma.
2. Studies that applied ML techniques using structured/tabular data (e.g., demographic data, clinical features, OCT measurements, visual field (VF) data).
3. Articles published in peer-reviewed journals between 2010 and 2024.
4. Studies published in English.

The exclusion criteria were as follows:

1. Book chapters, conference proceedings, abstracts, editorials, and gray literature.
2. Studies that used image data (e.g., fundus photographs or OCT images) without structured/tabular data.
3. Non-English publications.
4. Reviews, systematic reviews, or meta-analyses.
5. Studies focused solely on glaucoma progression, treatment outcomes, or unrelated comorbidities.
6. Duplicate records or studies without accessible full text.

### Study selection

**Fig. 1:**
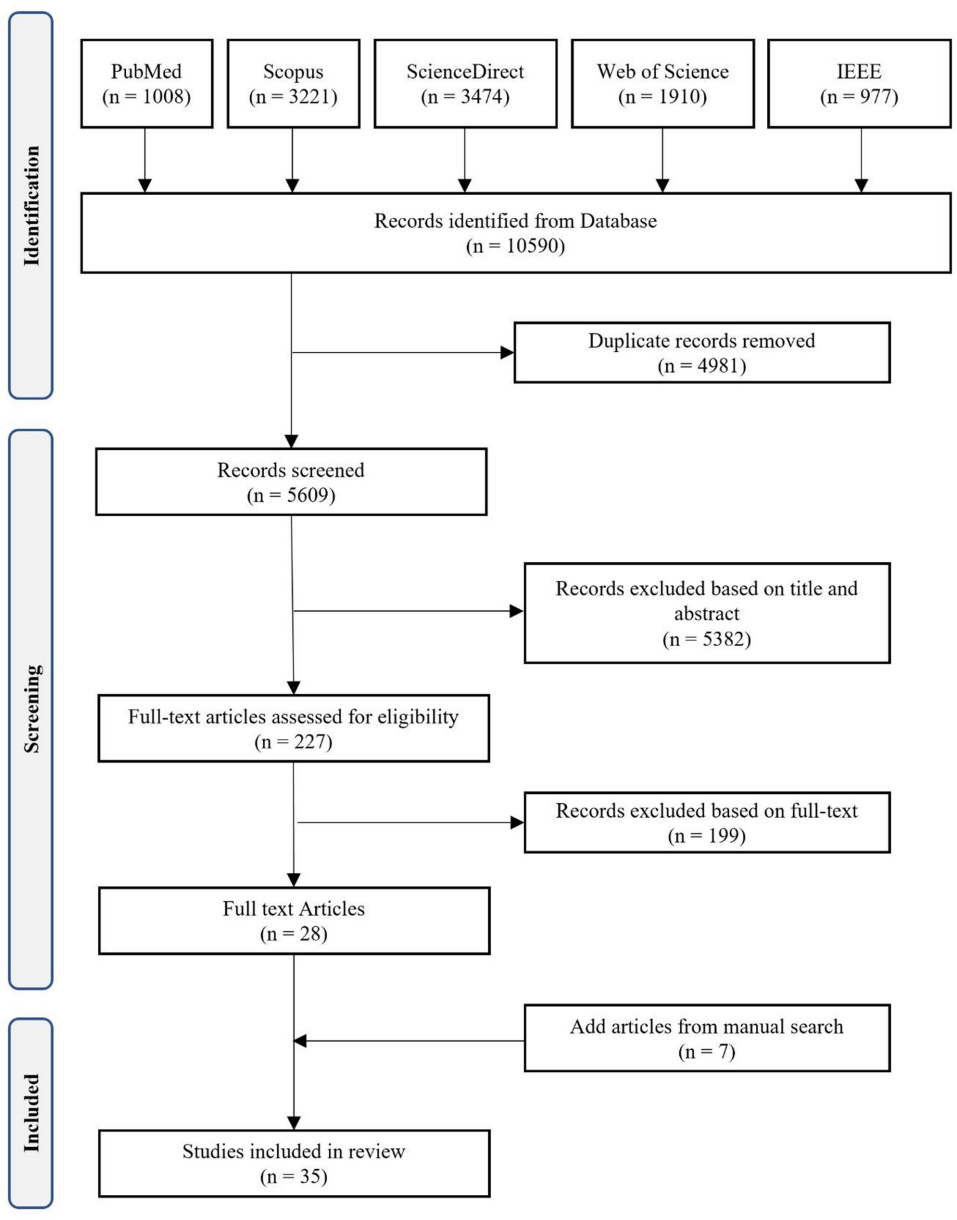
Flowchart of the study selection process (PRISMA)

The study selection followed the preferential reporting items for systematic reviews and meta-a nalyses (PRISMA) 2020 protocol [38]. After searching and retrieving articles from the five databases mentioned above, articles that met the eligibility criteria were imported into Endnote software, and duplicate articles were removed using this software (Table 1). The initial search resulted in 10,590 articles, of which 4981 duplicates were removed. Next, the titles and abstracts of 5609 articles were reviewed by two authors (M.S. and F.A.), and 5382 articles were further excluded. The full text of the remaining 227 articles was reviewed by two authors (M.S. and F.A.), and 28 articles were selected. Seven additional articles were included after reviewing the references to the selected articles. In total, 35 articles were included for analytical review (Fig. 1).

**Table 1:** Search queries

**Pubmed:** (Glaucoma[Title/Abstract]) AND ((Artificial intelligence[Title/Abstract]) OR (Machine learning[Title/Abstract]) OR (Deep learning[Title/Abstract]) OR (Decision support[Title/Abstract]) OR (Decision aid[Title/Abstract]) OR (Computer assisted[Title/Abstract])))
**SCOPUS**: TITLE-ABS-KEY (glaucoma) AND (TITLE-ABS-KEY (“Artificial intelligence”) OR TITLE-ABS-KEY (“Machine learning”) OR TITLE-ABS-KEY (“Deep learning”) OR TITLE-ABS-KEY (“Decision support”) OR TITLE-ABS-KEY (“Decision aid”) OR TITLE-ABS-KEY (“computer assisted”)) AND (LIMIT-TO (LANGUAGE, “English”))
**Web Of Sciemces**: (TS=(Glaucoma)) AND TS=(“Artificial intelligence” OR “Machine learning” OR “Deep learning” OR “Decision support” OR “Decision aid” OR “computer assisted”)
**SCIENCE DIRECT**: (Glaucoma) AND (“Artificial intelligence” OR “Machine learning” OR “Deep learning” OR “Decision support” OR “Decision aid” OR “computer assisted”)
**IEEE**: (“All Metadata”:Glaucoma) AND (“All Metadata”:“Artificial intelligence” OR “All Metadata”:“Machine learning” OR “All Metadata”:“Deep learning” OR “All Metadata”:“Decision support” OR “All Metadata”:“Decision aid” OR “All Metadata”:“computer assisted”)

### Data extraction and synthesis

Data extraction was performed independently by two authors (M.S. and F.A.), and any disagreements were resolved through discussion and consultation with the third author (H.M.). For each study, the following information was extracted: first author, year of publication, study design, sample size, data sources, data imbalance technique, variables, ML methods, performance metrics (e.g., AUC, accuracy, sensitivity, specificity), and key findings.

### Quality assessment

The methodological quality of the included studies was assessed using the QUADAS-2 tool [39]. The seven ordinal QUADAS-2 items, showed the quality of included study based on risk of bias and applicability concerns, were extracted for each studies. Each study was assessed as having a ‘low’ (all question answered yes), ‘high’ (at least one answered no) or ‘unclear’ (one answer unclear) risk of bias (four domain) and applicability concerns (three domain).

## Results

A total of Thirty-five studies were ultimately selected according to the eligibility criteria. In line with the objectives of this research, each study was monitored based on its aim, database, variables, number of records, ML methods, model performance indicators, and results. Table 2 summarizes the studies related to ML-based glaucoma diagnosis. This section summarizes the findings, focusing on the types of data used, the ML models employed, and the reported performance metrics.

### The methodological quality of included studies

**Fig. 2:**
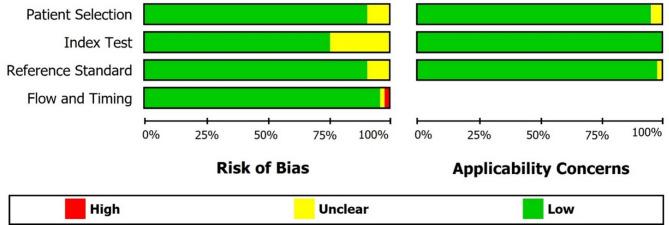
QUADAS-2 risk of bias and applicability concerns graph on each domain presented as percentages across included studies

The QUADAS-2 checklist of the 35 included studies is presented in Sup 1. According to the QUADAS-2 checklist, there was no severe bias in all studies, regarding the flow and timing, index test, and reference standard. Only one study had a high risk of bias related to patient selection [40]. Also, there were no severe concerns regarding applicability. The results obtained from the QUADAS-2 tool are shown in Fig. 2.

### Database characteristics and diversity of data types

Reviews show that diverse data from clinical examinations, paraclinical examinations, patient records, and demographic data have been used for ML modeling. Clinical examinations include refraction and BCVA, slit lamp, tonometry, gonioscopy, and ophthalmoscopy [41–47]. Paraclinical examinations include two categories: structural and functional eye examinations. Structural examinations include OCT, optical coherence tomography angiography (OCTA), pachymetry, corneal biomechanics, and fundoscopy [36, 48–54]. Functional examinations, including VF testing, including mean deviation (MD) and pattern standard deviation (PSD) parameters, were commonly analyzed [35, 37, 52, 55–60]. Patients’ Demographic data include age, gender, and race, along with patient history data, including systemic diseases (hypertension, diabetes, atherosclerosis, etc.), family history, other eye diseases (cataract, age-related macular degeneration (AMD)), medication history, and body mass index (BMI). These data are part of the database in some studies [35, 41, 43, 48, 54, 57].

**Fig. 3:**
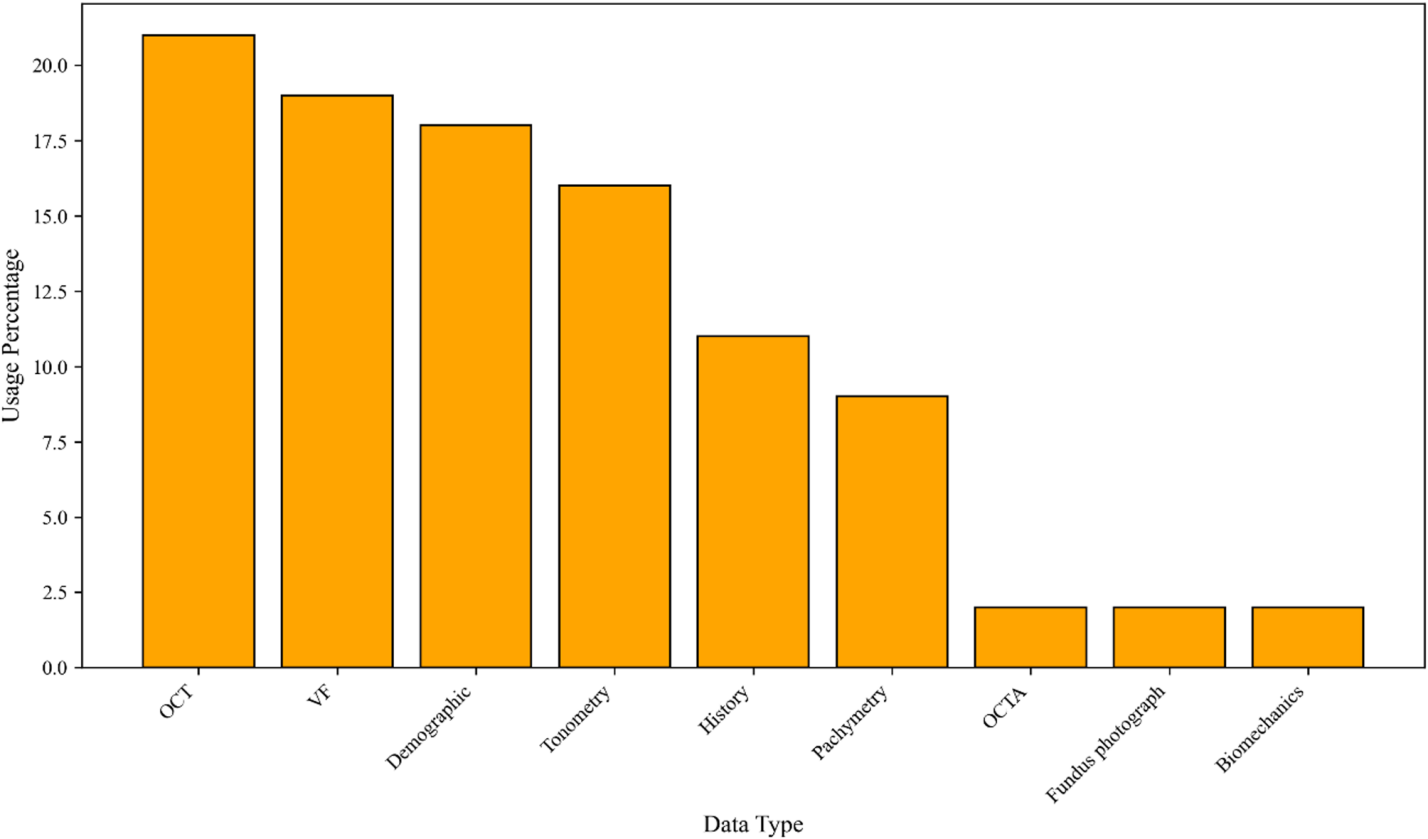
Variety of usage data in studies to diagnose glaucoma with ML

Some studies have modeled ML solely based on data from a single diagnostic method (e.g., data from OCT) to diagnose glaucoma [42, 46, 47, 56, 58, 60–63]. Other studies have modeled ML based on a combination of various data (e.g., combining demographic data, patient records, clinical examinations, etc.) [35–37, 40, 41, 43–45, 48–55, 57, 62, 64–70]. The most commonly used data types were OCT, VF, demographic, tonometry, pachymetry, and patient history, while OCTA, corneal biomechanics, and fundoscopy data were less frequently used (Fig. 3). The number of records in the study databases ranged from 100 to 33,611.

### Data preprocessing

Data preprocessing is crucial in ML studies as it enhances data quality and consistency, directly impacting the model’s performance and accuracy. Various data preprocessing techniques were employed in the studies, including segmentation techniques for extracting optic disc and cup regions in fundus images [46, 48, 51]and normalization to ensure features were on a comparable scale [36, 41, 53, 56, 66]. Handling missing data involved methods ranging from excluding features with significant missing values to using imputation techniques [47, 52, 67]. Addressing data imbalance was another critical point, with some studies utilizing techniques such as Synthetic Minority Over-sampling Technique (SMOTE) to balance datasets by generating synthetic samples [41, 49, 59]and class weight adjustment to handle imbalance by adjusting the weights of classes in the loss function [42, 43, 55]. Feature selection, crucial for improving model performance, included statistical tests like t-tests and ANOVA to identify significant features [35, 48, 49, 64]as well as algorithmic approaches such as recursive feature elimination and feature importance from tree-based models [41, 47, 52, 56, 65].

### ML methods

**Fig. 4:**
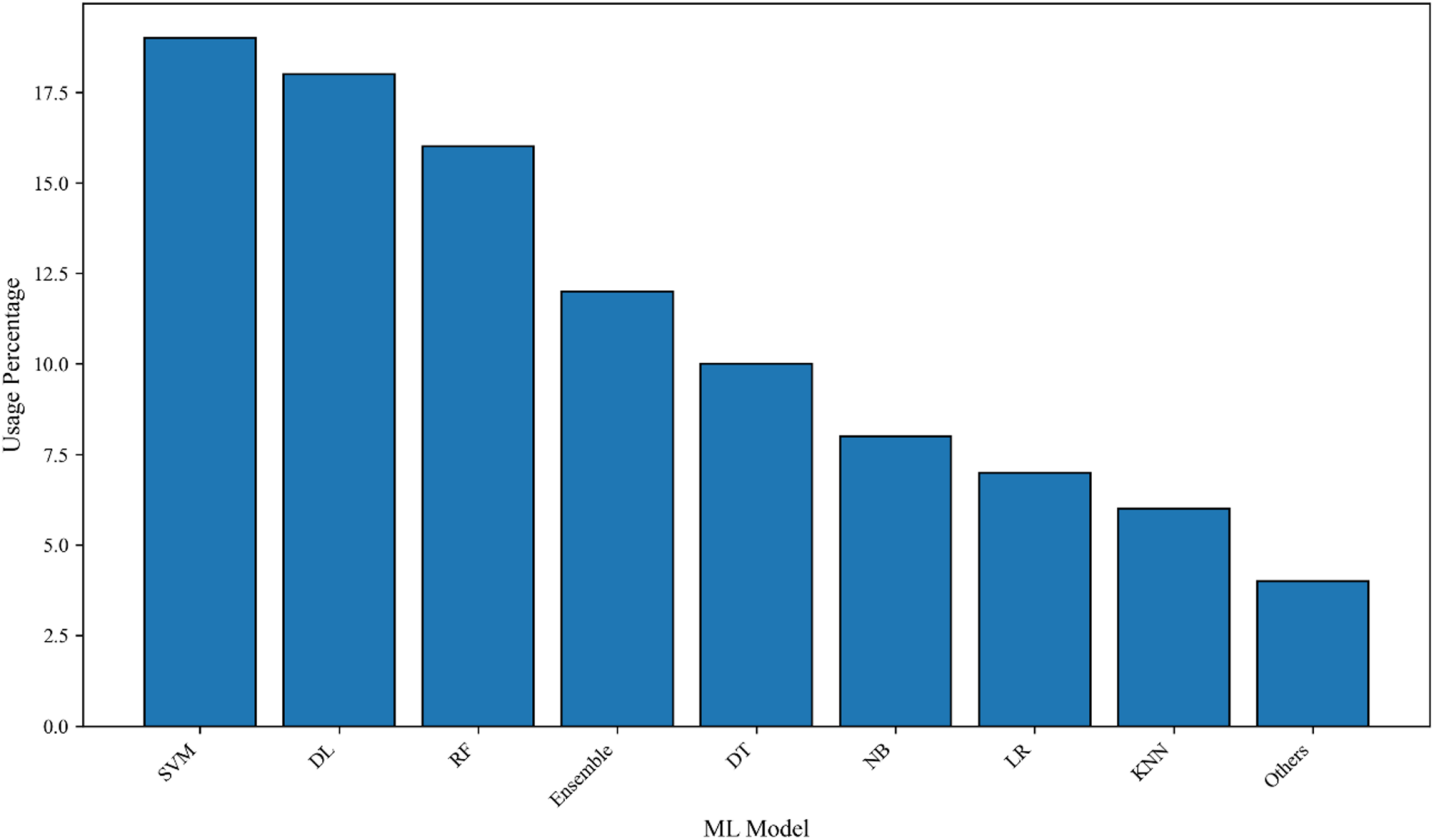
The ratio of using ML methods in glaucoma diagnosis. DL = deep learning; SVM = support vector machine; RF = random forest; DT = decision tree; LR = logistic regression; NB = Naïve Bays; KNN = k-nearest neighbors; Ensemble = XGBoost, AdaBoost, Gradient Boosting, and Soft Voting

Various ML methods were employed. The most commonly used supervised ML methods include support vector machine (SVM), DL [artificial neural network (ANN) and multilayer perceptron (MLP)], random forest (RF), Ensemble methods (XGBoost, AdaBoost, Gradient Boosting, Soft Voting), decision tree (DT), logistic regression (LR), naïve bays (NB), and k-nearest neighbors (KNN). Less frequently used methods include partial least squares discriminant analysis (PLS-DA), linear discriminant analysis (LDA), stochastic gradient descent (SGDC), radial basis function network (RBF), and relevance vector machine (RVM) (Fig. 4). Only one study used the unsupervised ML method K-means to create a glaucoma grading system based on VFs [42].

### Hyperparameter selection and validation methods

Hyperparameter tuning and robust validation were crucial elements in many studies. Commonly used methods for hyperparameter tuning included grid search and random search [36, 40, 44, 45, 67, 68]. Techniques such as k-fold cross-validation and leave-one-out cross-validation were employed to ensure robust performance evaluation [35, 37, 42, 52, 54, 57, 61].

### Performance metrics

In most studies, private databases were used to train and test the methods. External validation of the ML model using a secondary database was performed exclusively in the study by Li et al. [36]. The performance metrics for the ML methods used in the studies include accuracy, sensitivity, specificity, F1-measure, recall, and precision. Additionally, some studies used Area Under the Receiver Operating Characteristic Curve (AUC) to evaluate model performance. The accuracy metric in the studies ranges from 76 to 98.3%, sensitivity from 52 to 100%, specificity from 56 to 98%, AUC from 52.5 to 99%, and the precision index has been reported from 97 to 98%.

**Table 2:** Studies related to glaucoma diagnosis based on machine learning

Reference, Year	Purpose	Dataset	Number of records	Variables	Methods	Performance measure
Li, et al. 2023 [36]	To externally validate ML models for glaucoma detection from OCT data across different ethnicities.	Train dataset: 514 Asians (257 glaucoma/257 controls) Validation Dataset: 356 Asians (183 glaucoma/173 controls) and 138 Caucasians (57 glaucoma/81 controls)	514	11 variables: Age, Gender, Race, Fovea distance, Fovea angle, Optic disc area, Optic disc ratio, Optic disc orientation, Retinal vessel density, Spherical equivalent refractive error, Global RNFL thickness	Combination of four different ML models: LR, SVM, RF, and GB, using SVE.	Internal testing Asian Dataset: AUC = 0.96 External Testing Caucasian dataset: AUC = 0.93
Raju, et al. 2023 [35]	To develop a predictive analytic framework for the early prediction of glaucoma using electronic health records from over 650 hospitals and clinics across the USA	A balanced dataset containing: 33,611 patient records (16,805 glaucoma patients and 16,806 non-glaucoma patients)	33,611	32 binary variables: Gender, Cataract, Type_one, Type_two, Hypertension, Hypotension, Atherosclerosis, Lipoid_metabolism_disorder, Ischemic_heart_disease, Obesity, Lacrimal_disorder, Multivitamins, Steroids, Aspirin, Allopurinol, Atropine, Bacitracin, Chloramphenicol, Gentamicin, Gramicidin, Phenylephrine, Polymyxin, Sulfacetamide, Abnormal_glucose > 150, Bmi_high > 25, Glaucoma, African_american, Asian Caucasian, Hispanic, Native_american, Others	The XGBoost, MLP, and RF, LR	LR Accuracy = 0.72 RF Accuracy = 0.80 XGB Accuracy = 0.81 MLP Accuracy = 0.81
Ibrahim, et al. 2022 [49]	To propose a novel decision support system based on DL to diagnose glaucoma	337 patients (93 glaucoma instances and 244 normal instances)	337	Three attributes: Intraocular pressure, corneal thickness, and VF	DL	Accuracy = 0.98 Recall = 0.97 Precision = 0.98 false positive rate = 0.01 F1-measure = 0.98
Akter et al., 2022 [57]	to facilitate current diagnostic assessment of glaucoma by analyzing multiple features and introducing a new cross-sectional ONH feature from OCT images	Train dataset: 200 patients (100 normal subjects and 100 glaucoma) Test dataset: 55 patients (25 glaucomatous and 30 normal)	200	Structural features: Optic nerve head damage, Inner macular thinning, Thinning of the circumpapillary retinal nerve fiber layer (cpRNFL), Increased cup to disk ratio (CDR), Central corneal thickness (CCT) Functional features: Mean deviation (MD), Pattern standard deviation (PSD), Visual field index (VFI) Demographic features/clinical risk factors: Age, gender, ethnicity, Family history, Intraocular pressure (IOP), Refractive error, High or low blood pressure, Diabetes, previous eye injury	DL (pre-trained models; ResNet18 and VGG16), LR	DL: AUC = 0.98 Sensitivity = 1 Specificity = 0.96 Accuracy = 0.97 LR: AUC = 0.97 Sensitivity = 1 Specificity = 0.91 Accuracy = 0.95
Kooner, et al. 2022 [48]	To establish OCT/OCTA parameter ranges for healthy and glaucomatous eyes in a North Texas-based population and to develop an ML tool for identifying the most accurate diagnostic parameters for clinical glaucoma diagnosis	1371 eligible eyes; 462 Healthy eyes and 909 Glaucomatous eyes (377 ocular hypertension, 160 mild, 156 moderate, 216 severe), from 735 subjects.	1371	64 features: age, sex, race, family history of glaucoma, hypertension, diabetes, IOP, CCT, refractive error prior to any surgical correction, and **54 OCT/A parameters**: Foveal avascular zone (FAZ), FAZ area, FAZ perimeter, Flow density area density, Flow density length density, Cup area, Cup volume, Cup to disc ratio, Horizontal cup to disc ratioVertical cup to disc ratio, Rim area, Rim volume, Optic nerve head volume, Vessel density whole image, Vessel density inside disc, Vessel density peripapillary, Vessel density superior hemisphere, Vessel density inferior hemisphere, Temporal superior vessel density, Temporal inferior vessel density, Inferior temporal vessel density, Inferior nasal vessel density, Nasal inferior vessel density, Nasal superior vessel density, Superior nasal vessel density, Superior temporal vessel density, Retinal vessel density superior, Retinal vessel density inferior, Retinal vessel density temporal, Retinal vessel density nasal, RNFL thickness nasal superior, RNFL thickness superior nasal, RNFL thickness temporal inferior, RNFL thickness inferior temporal, RNFL thickness inferior nasal, RNFL thickness nasal inferior, RNFL thickness superior temporal, RNFL thickness temporal superior, RNFL thickness superior, RNFL thickness nasal, RNFL thickness inferior, RNFL thickness temporal, RNFL thickness peripapillary, RNFL thickness superior hemisphere, RNFL thickness inferior hemisphere, Average retinal full thickness, Retina full thickness inferior, Retina full thickness superior, Retinal full thickness temporal, Retina full thickness nasal, Retina inner thickness inferior, Retina inner thickness superior, Retina inner thickness temporal, Retina inner thickness nasal	Models were trained to solve a two-class problem (HE vs. GE) and four-class problem (HE vs. mild vs. moderate vs. severe GE). Six ML algorithms were compared using classical and DL approaches: XGBoost, DL, DT, SVM, PLSDA, and RF	XGBoost achieved the highest test performance: two-class: F1 score = 0.83 Accuracy = 0.83 F1 score = 0.62 Accuracy = 0.71
Wu, et al. 2022 [55]	To analyze the diagnostic capability of Spectralis OCT parameters on glaucoma detection using the SVM classification method in the population	752 eyes: 254 Normal eyes and 498 Glaucoma eyes	752	251 features: Nine feature groups: AGR (age, gender, refraction; 3 features), minimum rim width (MRW: T, TI, NI, N, NS, TS, G; 7 features), peripapillary nerve fiber layer thickness (ppNFLT: T, TI, NI, N, NS, TS, G; 7 features), retinal average thickness (RAT: T1, T2, I1, I2, N1, N2, S1, S2, C in 1, 3, 6 mm ETDRS grid; 9 features), nerve fiber layer (NFL: T1, T2, I1, I2, N1, N2, S1, S2, C in 1, 3, 6 mm ETDRS grid; 9 features), ganglion cell layer (GCL: T1, T2, I1, I2, N1, N2, S1, S2, C in 1, 3, 6 mm ETDRS grid; 9 features), inner plexiform layer (IPL: T1, T2, I1, I2, N1, N2, S1, S2, C in 1, 3, 6 mm ETDRS grid; 9 features), retinal average thickness in 8 8 grid (RAT 8 8; 64 features), and OCT (all OCT features; 114 features)	SVM	AUC = 0.84
Kaskar, et al. 2022 [41]	To model and evaluate classifiers for predicting self-reported glaucoma using a single ocular feature and non-ocular features	NIH Age-Related Eye Disease Study (AREDS) database 337 glaucomatous and 2,678 non-glaucomatous	3015	intraocular pressure (IOP) OU, age, gender, race, body mass index, systolic and diastolic blood pressure, and comorbidities (diabetes, arthritis, and AMD)	SVM, LR, and ADA	Sensitivity = 0.52 to 0.60 Specificity = 0.69 to 0.77
Huang, et al. 2022 [42]	To develop an objective and easy-to-use glaucoma staging system based on VFs using unsupervised ML to identify different severity levels of glaucoma	Train Dataset: 13,231 VFs from 8077 subjects Test Dataset: 8024 VFs from 4445 subjects	13,231	52 VF test locations	unsupervised k-means algorithm	Accuracy = 0.94
Huang, Jin et al. 2022 [56]	To propose an AI system to provide adequate assessment of glaucoma patients by developing a fine-grained grading DL system to evaluate VF loss compared to ophthalmologists and discuss the relationship between structural and functional damage for comprehensive evaluation of glaucoma level	16,356 (VFs)	16,356	Values of VFs as vector	DL	Accuracy = 0.85 to 0.90 AUC = 0.90 to 0.93
Leite, et al. 2021 [51]	To test and analyze different supervised ML models for the detection and classification of ophthalmic diseases (Glaucoma, high myopia, and low myopia) based on Corvis ST data	331 samples classified as: Normal (53), Low Myopia (69), High Myopia (106), and Glaucoma (103)	331	41 variables: Age, IOP [mmHg], Pachy [µm], Def. Amp. Max [mm], A1 Time [ms], A1 Velocity [m/s], A2 Time [ms], SSI, A2 Velocity [m/s], HC Time [ms], Peak Dist. [mm], Radius [mm], A1 Deformation Amp. [mm], HC Deformation Amp. [mm], A2 Deformation Amp. [mm], A1 Deflection Length [mm], HC Deflection Length [mm], A2 Deflection Length [mm], A1 Deflection Amp. [mm], HC Deflection Amp. [mm], A2 Deflection Amp. [mm], Deflection Amp. Max [mm], Deflection Amp. Max [ms], Whole Eye Movement Max [mm], Whole Eye Movement Max [ms], PachySlope [µm], DA Ratio Max (1 mm), ARTh, bIOP Integrated Radius [mm^-1], SP A1, A1 Deflection Area [mm²], HC Deflection Area [mm²] A2, Deflection Area [mm²] A1, dArc Length [mm], HC dArc Length [mm], A2 dArc Length [mm], dArcLengthMax [mm], Max InverseRadius [mm^-1], DA Ratio Max (2 mm), CBI	10 ML models: RF, NB, SVM, MLP, DT, GB, KNN, SGDC, ADA and LR.	GBC result: Accuracy = 0.76 AUC = 0.52 Recall = 0.78 F1-score = 0.70
Escamez, et al. 2021 [64]	To develop a classifier to differentiate between healthy and early-stage glaucoma eyes based on peripapillary RNFL thicknesses measured with OCT using ML algorithms with high interpretability	90 eyes with early-stage glaucoma and 85 controls	175	25 variables: Baseline characteristics: age, gender, VA, SE, IOP, VFI, SAP MD, SAP PSD RNFL thickness measures: Average, 1 to 12 CH, IQ, SQ, TQ, NQ VA: Visual acuity; SE: Spherical quivalent; D: Diopters; IOP: Intraocular pressure; VFI: Visual field index; SAP: Standard automated perimetry; MD: Mean deviation; PSD: Pattern standard deviation. aUnpaired *t*-test; b*χ*2 test; cKrusal-Wallis test. CH: Clock-hour; IQ: Inferior quadrant; SQ: Superior quadrant; TQ: Temporal quadrant; NQ: Nasal quadrant.	GB and DT	Accuracy = 0.89 AUC = 0.95 Sensitivity = 0.89 Specificity = 0.88
Omar, et al. 2021 [40]	To propose a new model for mass screening of glaucoma aimed at decreasing the false negative rate by applying nine different ML techniques in a majority voting model and building a consensus ensemble from the top five techniques with the highest accuracy	202 records of non-glaucoma cases and 297 of glaucoma cases (primary open angle glaucoma (POAG) or normal tension glaucoma (NTG)).	499	7 features: age, ocular pressure, cornea thickness, retinal nerve fiber layer (RNFL) thickness, glaucoma hemifield test (GHT), history of macular degeneration (MD), and pattern standard deviation (PSD).	Different ML techniques: SVM, NB, KNN, RF, DT and LR	Ensemble of the top five performing techniques, which included LR, RF, SVM, KNN, and DT Accuracy = 0.90 to 0.83
Oh, et al. 2021 [37]	To develop a ML prediction model for glaucoma diagnosis and an explanation system for a specific prediction	975 eyes (of 430 patients) with glaucoma (POAG (primary open angle glaucoma) or NTG) and 649 eyes (of 377 patients) without glaucoma.	1624	22 features and a class label: Sex, Age, GHT, VFI, MD, Pattern standard deviation, RNFL superior, RNFL Nasal, RNFL inferior, RNFL temporal, Mean of RNFL thickness, Intraocular pressure, Cornea thickness, BCVA, Spherical equivalent, Axial length, Neuro-retinal rim, Cup, Disc, Mean of cup/disc, vertical cup/disc ratio, CNN degree (In the case of fundus images, we build a prediction model based on a convolutional neural network (CNN))	ML models: SVM, C5.0, RF, and XGboost	Accuracy = 0.90 to 0.94 **XGboost** AUC = 0.94
Sharifi, et al. 2021 [43]	To develop a ML model for predicting glaucoma and identifying its risk factors	5190 people, including 4561 non-glaucoma participants and 87 glaucoma patients.	5190	67 demographics, ophthalmologic, optometric, perimetry, and biometry features. Sex, Body Mass Index (in kg/m2), Socioeconomic Status, Smoking, Diabetes Drug, Systolic Blood Pressure (in mm/Hg), Hyper Tension Drug, Visual Accuity* (in logMAR), Hyperopia, Glaucoma Drug*, Glaucoma*, Astigmatism* (in Diopter), Angle Closure*, Vertical Cup to Disk Ratio*, Anterior Chamber Depth*(mm), Lens Thickness* (in mm), Corneal Radius, Flat* (in mm), Keratometry* (K1), Iris Barycentric-X-Coordinate*, Pupil Barycentric-XCoordinate*, Pupil Distance*(in mm), Age (in Years), Occupation Status, Marital Status, Diabetes, Iris Color*, Diastolic Blood Pressure (in mm/Hg), Intraocular Pressure* (in mm/Hg), Myopia*, Nuclear Cataract*, Cortical Cataract*, Posterior Subcapsular Cataract*, Spherical Equivalent*(in Diopter), Number of Visual Detect*, Axial length* (in mm), Corneal Thickness *(in mm), Corneal White to White Diameter*, (in mm), Corneal Radius, Steep* (in mm), Keratometry* (K2), Iris Barycentric-Y-Coordinate*, Pupil Barycentric-Y-Coordinate* *Stars show that the feature has been measured in both eyes.	ML models: DT, KNN, SVM, RF, ET and BAG.	Ensemble method: Accuracy = 0.83 Sensitivity = 0.82 Specificity = 0.81 AUC = 0.88
Rabiolo, et al. 2021 [50]	To compare the ability of peripapillary and macular structural parameters, vascular parameters, and their integration to discriminate among glaucoma patients, glaucoma suspects, and healthy controls	196 eyes of 119 patients includes: • glaucoma (*n* = 81) • glaucoma suspects (*n* = 48) • healthy controls (*n* = 67)	196	35 variables: Age (y), Caucasian, Male/female, Right eye/left eye, SS nerve, SS macula, VF MD (dB), Disc area (mm2), Rim area (mm2) **OCTA**: pRNFL thickness (µm): Average, Superior quadrant, Nasal quadrant, Inferior quadrant, Temporal quadrant, Perfusion Density (%): Average, Superior quadrant, Nasal quadrant, Inferior quadrant, Temporal quadrant **OCT**: GCIPL thickness (µm): Average, Supero-temporal, Superior, Supero-nasal, Infero-temporal, Inferior, Infero-nasal Perfusion Area (%): Average, Supero-temporal, Superior, Supero-nasal, Infero-temporal, Inferior, Infero-nasal	Elastic net regression and three supervised ML classifiers (RF, SVM, NB).	AUC = 0.57 to 0.90
Eswari, et al. 2021 [44]	To design an intelligent prediction system to predict glaucoma among diabetic patients using a ML model	100	100	29 variables	SVM	AUC = 0.83 Accuracy = 0.97 Sensitivity = 0.94 Specificity = 0.95
Lee, et al. 2021 [58]	To propose an improved ML-based glaucoma diagnostic model using VF clustering to reflect structural-functional patterns of glaucoma	Visual field testing data of 375 healthy and 257 glaucomatous eyes of Korean subjects aged from 13 to 82 years	632	**65 variables** derived from the Garway-Heath map and the glaucoma hemifield test (GHT) sector map were included in the input variables in addition to the 52 SAP visual filed locations	Four classifiers: LDA, NB, SVM, and ANN	AUC = 0.91
Lu, et al. 2021 [65]	To apply ML methodologies on ocular biomechanical data to improve glaucoma classification accuracy, focusing on IOP and biomechanical behaviors as early indicators of glaucoma	52-patient biomechanical dataset that includes 20 glaucoma (40 eyes) and 32 healthy subjects (64 eyes).	104	5 variables: IOP, Corneal radius, Corneal thickness, Corneal stiffness, Corneal modulus	LR, SVM, RF and GB	Logistic regression: Accuracy = 0.98 AUC = 0.99 Sensitivity = 0.98 Specificity = 0.97
Lazouni, et al. 2019 ^45^	To develop an intelligent computer-aided diagnosis system able to detect glaucoma at its early phase	1415 eyes of 723 patients (467 are healthy, and 256 are glaucoma patients).	1415	17 variables: 1 The VF, 2 Intraocular pressure with aplanation tonometer, 3 Intraocular pressure with air jet tonometer, 4 The gonioscopy, 5 The measure of cup – to disc ratio, 6 The corneal thickness (CT), 7 The correction of the intraocular pressure with aplanation tonometer using the pachymetry, 8 The correction of the intraocular pressure with air jet tonometer using the pachymetry, 9 Family background, 10 Measurement of the anterior chamber depth using echography, 11 Measurement of the anterior chamber depth using topography, 12 Axial length, 13 Visual acuity with correction, 14 Visual acuity without correction, 15 Sex, 16 Age, 17 The eye	ANN, SVM, KNN and DT	SVM: Accuracy = 0.94
Shigueoka, et al. 2018 [66]	To evaluate the ability of ML classifiers using OCT and SAP parameters to discriminate between healthy and glaucomatous individuals and compare it to the diagnostic ability of the combined structure-function index (CSFI), general ophthalmologists, and glaucoma specialists	58 eyes of 58 patients with early to moderate glaucoma and 66 eyes of 66 healthy individuals.	124	18 variables: Age, Left eye, Female gender, Ethnicity, VA, SE, IOP, Medications, SAP MD, SAP PSD, FDT MD, FDT PSD, SAPrgc, OCTrgc, WRGC, CSFI, Glaucoma specialist likelihood scale, General ophthalmologist likelihood scale	10 ML classifiers: BAG, NB, MLP, RBF, RF, ENS, CTREE, ADA, Linear SVM and Gaussian SVM	AUC = 0.80 to 0.94 Sensitivity = 0.51 to 0.83 Specificity = 0.90
Kim, et al. 2017 [52]	To develop ML models with strong prediction power and interpretability for the diagnosis of glaucoma based on RNFL thickness and VF parameters	297 cases of eyes with glaucoma and 202 cases of eyes without glaucoma	499	12 variables: Gender, age, ocular pressure, cornea thickness, RNFL SUP, RNFL NAS, RNFL INF, RNFL TMP, VFI, MD, PSD, GHT	Four ML algorithms: C5.0, RF, SVM, and KNN.	RF: Accuracy = 0.98 Sensitivity = 0.98 Specificity = 0.97 AUC = 0.97
Wyawahare and Patil, 2016 [46]	To utilize ML classifiers for automatic discrimination between healthy and glaucomatous eyes based on structural optic nerve head measurements in fundus images	NA	NA	6 variables: cup to disc ratio, horizontal cup diameter, vertical cup diameter, rim area, horizontal rim width, vertical rim width	Eight ML classifiers: LR, NB, Linear and Quadratic discriminant analysis, discriminant analysis with Mahalanobis function, SVM, MLP and ANN	AUC = 1
Asaoka, et al. 2016 [59]	To differentiate the VFs of preperimetric OAG patients from the VFs of healthy eyes using a DL method	171 preperimetric glaucoma VFs from 53 eyes in 51 OAG patients and 108 healthy eyes of 87 healthy participants.	279	3 variables: total deviation (TD), mean deviation (MD), and pattern standard deviation (PSD)	Deep Learning Classifier (a **deep feed-forward neural network**, along with other ML methods, including RF, gradient boosting, SVM, and **ANN**)	AUC = 0.92
Yoo, et al. 2015 [67]	To differentiate the VFs of preperimetric OAG patients from the VFs of healthy eyes using a deep learning method	4113 participants: 386 suspected glaucoma and 3727 healthy.	4113	9 variables: Four non-ophthalmologic factors (sex, age, menopause, and duration of hypertension) and five ophthalmologic factors (IOP, spherical equivalent refractive errors, vertical cup-to-disc ratio, presence of superotemporal retinal nerve fiber layer [RNFL] defect, and presence of inferotemporal RNFL defect).	ANN	AUC = 0.89 Accuracy = 0.84 Sensitivity = 0.78 Specificity = 0.85
Asaoka, et al. 2014 [60]	To compare the VFs of preperimetric OAG patients with the VFs of healthy eyes and to discriminate these two groups using the RF machine-learning method	171 from OAG or OAG suspect patients and 108 healthy eyes. Clinical records at the University of Tokyo Hospital.	279	54 variables: 52 Total Deviation (TD) values, Mean Deviation (MD), and Pattern Standard Deviation (PSD)	RF	AUC = 0.79
Yoshida, et al. 2014 [61]	To diagnose glaucoma based on SD-OCT measurements using the ‘RF’ method	126 eyes of 126 OAG patients and 84 eyes of 84 normal subjects	210	151 OCT parameters: Thickness measurements of circumpapillary retinal nerve fiber layer (cpRNFL), the macular RNFL (mRNFL) and the ganglion cell layer-inner plexiform layer combined (GCIPL)	RF	AUC = 0.98
Vidotti, et al. 2013 [53]	To investigate the sensitivity and specificity of ML classifiers and SD-OCT for the diagnosis of glaucoma	62 glaucoma patients and 48 healthy individuals	110	20 variables: SAP parameters included in the analysis were MD, PSD, and GHT. The OCT technology provides RNFL thickness maps with 17 parameters: average thickness, 4 quadrants (superior, inferior, nasal, and temporal) and 12 clock hour measurements.	ML classifiers: BAG, NB, MLP, RBF, RF, ENS, CTREE, ADA, Linear SVM and Gaussian SVM	AUC = 0.77 to 0.94 Sensitivity = 0.82 to 0.93 Specificity = 0.56 to 0.83 Best: **RAN**
Barella, et al. 2013 [47]	To investigate the diagnostic accuracy of ML classifiers using RNFL and ONH parameters obtained with spectral domain OCT for the diagnosis of glaucoma	57 patients with early to moderate primary OAG and 46 healthy patients	103	23 parameters measured with the SD-OCT (17 RNFL and 6 ONH).	10 ML classifiers: BAG, NB, linear SVM, Gaussian SVM, MLP, RBF, RF, ENS, CTREE, and ADA	CTREE AUC = 0.68 to RAN 0.87
Sugimoto, et al. 2013 [68]	To develop a classifier to predict the presence of VF deterioration in glaucoma suspects based on OCT measurements using the ML method known as the RF algorithm	293 eyes of 179 participants with OAG or suspected OAG. (224 eyes were glaucomatous, and 69 eyes were normal)	293	9 variables: Age (years), MD (dB), AL (mm), m-RNFL (µm), cp-RNFL (µm), GCL + IPL (µm), Rim area (mm2), Eye (right/left), Gender (male/female)	RF	AUC = 0.90
Hatanaka, et al. 2012 [54]	To evaluate computerized glaucoma risk assessment using patients’ clinical information along with automated nerve fiber layer defects (NFLDs) detection method performances.	79 NFLDs in high risk group and 81 normal cases.	170	11 variables systolic and diastolic pressure, self-corrected visual acuity, spherical refraction, refraction axis, corneal curvature and thickness, intra ocular pressure, glade of angle, presence of PPA, and presence of NFLDs	ML classifiers: ANN, RBF, KNN, and SVM.	Sensitivity = 0.80 Specificity = 0.75 Accuracy = 0.78
Bizios, et al. 2011 [62]	To investigate the performance of data fusion methods and techniques for the combination of SAP and OCT data for glaucoma diagnosis using ANN	125 healthy persons and 135 patients with glaucomatous optic nerve heads	260	38 Fused OCT Parameters and 52 Fused SAP Parameters	ANN	AUC = 0.97
Boland, et al. 2011 [69]	To define and test a new approach to glaucoma diagnosis by combining quantitative data from imaging and VF tests using the anatomical organization of retinal ganglion cells, leading to the development of the Structure Function Index	1499 eyes of glaucoma suspects and 895 eyes with glaucoma	2394	4 variables: SAP indices: Total Deviation values, mean deviation (MD), Glaucoma Hemifield Test (GHT). optic nerve imaging using the Heidelberg Retina Tomograph (HRT): Rim area and the rim area predicted by Moorfields regression analysis (MRA) in each of the six sectors.	Statistical analysis	AUC = 0.78
Racette, et al. 2010 [70]	To investigate if combining optic disc topography and short-wavelength automated perimetry (SWAP) data improves the diagnostic accuracy of RVM classifiers for detecting glaucomatous eyes compared to using each test alone	144 glaucoma patients and 68 healthy controls	212	18 variables: Age (yrs, mean ± SD, range), Gender (% male), SWAP MD (Mean ± SD) (dB), SWAP MD Range (dB), SWAP PSD (Mean ± SD) (dB), SWAP PSD Range (dB), Optic disc area (mm2), Area below reference (cup area) (mm2), Mean height contour (mm), Height variation contour (mm), Volume below reference (mm3), Volume above reference (mm3), Cup shape, Mean cup depth (mm), Mean RNFL thickness (mm2), Reference height (mm), Rim area (mm), Rim-to-disc area ratio	RVM	AUC = 0.93
Bizios, et al. 2010 [71]	To compare the performance of two MLC, ANN and SVM, based on RNFL thickness measurements by OCT for glaucoma diagnosis and assess the effects of different input parameters	90 healthy persons and 62 glaucoma patients	152	values of the 256 A-scans of each OCT image into a set of six values that we then used as input data in our ML classifiers.	ANN and SVM	ANN AUC = 0.98 SVM AUC = 0.98
Huang, et al. 2010 [63]	To determine whether LDA and ANN can improve the differentiation between glaucomatous and normal eyes in a Taiwan Chinese population based on RNFL thickness measurement data from scanning laser polarimetry with variable cornea compensation (GDx VCC)	79 glaucoma and 86 healthy subjects	165	14 variables: TSNIT average (total average RNFL thickness), superior average, inferior average, the TSNIT standard deviation, symmetry, superior ratio, inferior ratio, superior/nasal, maximum modulation, superior maximum, inferior maximum, ellipse modulation, normalized superior area, and normalized inferior area.	LDA and ANN	LDA AUC = 0.95 ANN AUC = 0.97

ML = Machine Learning; DL = deep learning; ANN = artificial neural network; SVM = support vector machine; RF = random forest; DT = decision tree; LR = logistic regression; NB = Naïve Bays; KNN = k-nearest neighbors; GB = gradient boosting; BAG = Bagging; SVE = soft voting ensembling; RVM = Relevance Vector Machine; RBF = radial basis function; MLP = multilayer perceptron; ENS = ensemble selections; CTREE = classification tree; ADA = AdaBoost; ET = Extra Trees; LDA = linear discriminant analysis; SGDC = Stochastic Gradient Descent; PLSDA = partial least-squares discriminant analysis; OAG = open angle glaucoma; VF = Visual Field; RNFL = retinal nerve fiber layer; ONH = optic nerve head

## Discussion

This systematic review critically examines the current landscape of ML applications in glaucoma diagnosis, highlighting significant diversity in data elements, ML methods, and performance metrics reported across 35 studies. Our findings emphasize the growing potential of ML to revolutionize glaucoma diagnosis, although further discussion on challenges and considerations is necessary.

### Database characteristics and diversity of data types

The reviews indicate that diverse data have been utilized for ML modeling. It should be noted that due to the multifactorial nature of glaucoma, making a diagnosis based on a single factor is challenging. VF examination may have limitations in sensitivity and specificity, potentially failing to detect early glaucomatous damage or producing false-positive results [3]. VF test results can also vary across examination sessions and be influenced by factors such as patient cooperation, learning effects, and fatigue [72]. OCT data is frequently utilized, providing critical insights into structural changes associated with glaucoma. Features such as RNFL thickness and optic disc parameters are essential for accurate prediction. But we must note that OCT does not directly assess visual function, and its measurements can be affected by subtle early-stage glaucoma changes, signal strength, patient cooperation, and image artifacts [3, 73–75].

Some others have considered combining two to three categories of data [35, 36, 50, 51, 53, 62, 69]. Additionally, in several other studies, a combination of diverse data was used to aggregate structural features, functional features, demographic information, and risk factors into a consolidated database [37, 43, 55, 57, 64, 66]. The use of demographic features (e.g., age, gender, ethnicity) and risk factors enhances model performance and ensures that findings are relevant across different populations.

Given that glaucoma is multifactorial, combining data from EHR to create a reliable ML model and integrating it into clinician workflow as a CDSS is preferable. This comprehensive approach, enhances the model’s accuracy and reliability, enabling informed decision-making and more effective management of glaucoma. Such integration advances personalized care and reduces glaucoma-related complications [16, 76–78].

As mentioned, the number of records in study databases ranges from 100 to 33,611. For example, in the study by Eswari et al. [44]modeling was performed on only 100 records. A few records in AI studies can create or exacerbate several problems [79–81]; ML models primarily operate based on training data, and a limited number of records can lead to a lack of training data, potentially causing model inefficiency and increasing the likelihood of overfitting. Insufficient data prevents ML models from learning general patterns and instead forces them to learn specific patterns, weakening their ability to generalize to new data. This issue is particularly significant in studies dealing with rare phenomena or occurrences, where a small number of records can prevent AI models from being robust enough to detect these rare phenomena.

### Data preprocessing

Data preprocessing is crucial in ML, particularly for handling data imbalance and feature selection. By addressing data imbalance using techniques like SMOTE or class weight adjustment, the model becomes more robust, leading to improved accuracy and better generalization across all classes. Hybrid and ensemble methods provide robust solutions but at the cost of increased complexity and computational demands. Selecting the appropriate method depends on the specific characteristics of the dataset and the application context [82–84]. Feature selection, on the other hand, helps in identifying the most relevant features, reducing dimensionality, and improving model performance by eliminating noise and irrelevant data. Together, these preprocessing steps enhance the reliability and effectiveness of machine learning models. Studies that include comprehensive feature sets (structural and demographic) generally report better performance.

### ML methods

OCT data, being highly detailed and quantitative, was predominantly used across various studies. Its high-resolution imaging capabilities make it particularly suitable for structural analysis of the optic nerve head and retinal layers. Studies leveraging OCT data often employed complex ML models like deep learning (DL) due to the rich feature set extracted from these images. For instance, studies utilizing DL models with OCT data reported high performance metrics, with AUC values frequently exceeding 0.90. This suggests that DL models are particularly adept at capturing the intricate patterns in OCT data, leading to superior diagnostic accuracy.

VF data provides functional information about the patient’s vision, which is crucial for diagnosing and assessing the progression of glaucoma. ML methods such as SVM and RF were commonly applied to VF data. These methods are well-suited for handling the non-linear and complex nature of VF datasets. Studies using VF data reported varied performance, with accuracy ranging from 0.76 to 0.94, indicating that while VF data is valuable, its integration with structural data (like OCT) can enhance the overall model performance.

Demographic features (e.g., age, gender, ethnicity) and clinical risk factors (e.g., IOP, and family history of glaucoma) were often combined with structural and functional data to provide a comprehensive dataset. This combination helps in developing models that are not only accurate but also generalizable across diverse populations. LR and ensemble methods (e.g., XGBoost, RF) were effectively used with such mixed datasets, achieving balanced performance metrics. For example, studies integrating demographic data with clinical measurements and OCT/VF data reported accuracies up to 0.90, highlighting the importance of a multifaceted approach in glaucoma diagnosis.

The trend in ML methods for glaucoma diagnosis includes the dominance of DL models, the integration of multimodal data for comprehensive diagnostics, and the push for explainable AI (XAI) to enhance model interpretability. Additionally, the use of big data, cloud computing, and edge computing is facilitating scalable, real-time, and accessible diagnostic solutions.

### Performance measures and model effectiveness

The studies reviewed frequently reported accuracy and AUC as primary performance metrics. Models trained on OCT data using DL techniques generally achieved the highest AUC values (often above 0.90), indicating excellent discriminative ability. In contrast, models using VF data alone showed more variability in performance, suggesting that structural data might provide more consistent features for ML models.

Sensitivity and specificity are crucial for evaluating the clinical utility of ML models. High sensitivity ensures that the model effectively identifies glaucoma cases, while high specificity reduces false positives. Studies incorporating balanced datasets and robust preprocessing techniques, like SMOTE for handling data imbalance, reported improved sensitivity and specificity, often exceeding 0.90. For example, a study that used SMOTE with VF and OCT data reported sensitivity and specificity of 0.97 and 0.96, respectively. This underscores the importance of addressing data imbalance to enhance model reliability.

### Feature selection and model interpretability

Feature selection methods, such as recursive feature elimination and feature importance from tree-based models, played a significant role in improving model performance. By selecting the most relevant features, these methods reduced model complexity and enhanced interpretability.

Studies employing feature selection techniques reported improved performance metrics, such as higher accuracy and AUC, compared to those using all available features. For instance, a study using recursive feature elimination with OCT data achieved an AUC of 0.95, demonstrating the effectiveness of this approach. The reported performance metrics varied widely, reflecting the diversity in methods and data elements. While many studies reported high-performance metrics, several challenges were identified.

• **Overfitting** is especially prevalent in studies with small sample sizes or highly complex models, leading to poor generalization of new data [48, 51, 59].

• **Data Variability**: Differences in data acquisition methods and patient demographics affected model performance, highlighting the need for standardized protocols [43, 46, 55, 58].

• **Interpretability**: Balancing the accuracy of ML models with their interpretability was a significant concern for clinical applications [41, 50, 59, 65].

### Generalizability concerns

A key concern is the generalizability of the ML models due to the Heterogeneity in datasets. Many studies relied on specific modalities, such as OCT and VF assessments, which may not be uniformly available across different clinical settings. This reliance on particular data types can limit the applicability of the findings to broader patient populations. To enhance generalizability, future studies should focus on incorporating diverse datasets that reflect a wider range of demographic and clinical characteristics. Also, datasets include diverse populations (e.g. American, Asians, Caucasians, and African), which is crucial for developing generalizable models. This diversity helps mitigate biases and ensures that models perform well across different demographic groups.

### Ethical considerations

As with any AI application in healthcare, the application of ML in glaucoma diagnosis involves several ethical considerations that must be carefully addressed to ensure the responsible use of technology in clinical practice. One of the primary concerns is data privacy and security. Given the sensitive nature of medical data, it is crucial to implement robust measures to protect patient information from unauthorized access and breaches. This includes using encryption, anonymization, and secure storage solutions to safeguard patient records. Another important ethical issue is the potential for bias and discrimination in ML models. ML algorithms trained on biased datasets can inadvertently perpetuate existing inequalities, leading to unfair treatment of certain patient groups. It is essential to ensure that training data is representative of the diverse patient population and to use techniques that can identify and mitigate bias in the models. Regular audits and validation studies should be conducted to monitor and address any biases that may arise. Transparency and explainability are also critical ethical considerations. Clinicians must be able to understand and interpret the outputs of ML models to make informed decisions about patient care. Therefore, it is important to develop models that are not only accurate but also interpretable. Providing clear explanations of how models arrive at their predictions can help build trust and facilitate the integration of ML tools into clinical workflows. Additionally, there are concerns about the accountability and liability associated with ML-driven diagnostic tools. In the event of an incorrect diagnosis or treatment recommendation, it is important to delineate the responsibilities of the ML developers, healthcare providers, and institutions. Establishing clear guidelines and regulatory frameworks can help manage these risks and ensure that ethical standards are maintained. Lastly, informed consent and patient autonomy must be respected. Patients should be made aware of how their data will be used in ML models and should have the option to opt-out if they choose. Transparent communication about the benefits and limitations of ML-driven diagnostics can help patients make informed decisions about their healthcare.

### 4.8 Limitations and future directions

Few studies have explored initial predictive models for the onset of glaucoma using EHR data obtained from multiple clinical centers. Future predictive models should focus on a large, diverse populations to bring substantial clinical value to ophthalmologists. According to Stagg et al., ML-based decision support systems should be integrated into the clinical practice enhance efficiency [16]. With the widespread adoption of EHR and its secondary use in research, there is an opportunity to apply retrospective data-driven approaches to develop predictive models by understanding comorbidities that affect glaucoma onset and related medications [85]. While several models have been developed to predict the onset and progression of glaucoma based on structural and functional eye data, but few have utilized systemic data from EHR [7, 67, 86–88]. Digitizing healthcare data presents a significant opportunity to better understand the complex relationships between systemic diseases and glaucoma [1, 89]. Understanding these relationships is crucial for the precise treatment of glaucoma patients, who are often older and have comorbid conditions such as hypertension and diabetes [90].

The reviewed studies faced several limitations, such as the retrospective nature of the databases, a lack of external validation, and potential selection bias. Future research should prioritize prospective studies with large, diverse cohorts to validate the generalizability of ML models across different populations and clinical settings. To facilitate the reproducibility and comparability of ML studies in glaucoma diagnosis, there is a need for standardized reporting guidelines and data-sharing practices. Collaboration among researchers, clinicians, and industry partners can help establish best practices and accelerate the translation of these models into clinical practice. More studies are needed to assess the practicality of implementing these ML models in clinical workflows, including considerations of time efficiency and clinician usability. Future studies should focus on standardizing data acquisition and preprocessing methods to reduce variability and improve the comparability of performance metrics across different populations and clinical settings.

## Conclusion

This systematic review has highlighted the significant advancements and potential of ML models in the diagnosis of glaucoma. Among the various ML models evaluated, DL models, have shown the most promise when applied to OCT data, achieving high accuracy and robust performance metrics such as AUC values frequently exceeding 0.90. SVM have also demonstrated effectiveness, especially when combined with functional data like VF test and demographic features. These findings underscore the importance of using diverse and comprehensive data sources to enhance the diagnostic capabilities of ML models.

Theoretically, this review contributes to the understanding of how different ML models and data types can be integrated to improve glaucoma diagnosis. It underscores the multifactorial nature of glaucoma and the need for models capable of analyzing complex and heterogeneous data. Practically, while ML integration into clinical workflows holds promise for earlier and more accurate glaucoma detection—potentially improving patient outcomes—it also presents several challenges. These include navigating regulatory approval processes, ensuring seamless integration with electronic medical records (EMRs), providing adequate training for clinicians, and addressing the need for model transparency and interpretability to gain clinical trust. Overcoming these barriers is essential for the successful real-world deployment of ML-driven decision support tools in ophthalmology.

This review has made several key contributions to the field; It provides a comprehensive analysis of the effectiveness of various ML models in diagnosing glaucoma. It highlights the importance of integrating multiple data types, such as OCT, VF, and demographic data, to enhance model performance. It identifies specific ML models, like DL and SVM, that have shown exceptional potential in this domain.

One of the primary practical advantages of using ML models in glaucoma diagnosis is the ability to process and analyze large volumes of data quickly and accurately. This can lead to early diagnosis and timely intervention, potentially slowing the progression of glaucoma and preserving vision. Additionally, ML models can assist in reducing the burden on clinicians by automating parts of the diagnostic process, allowing healthcare providers to focus on more complex and critical tasks. The use of ML can also facilitate personalized treatment plans by identifying specific patterns and risk factors unique to each patient.

Despite the promising findings, this review also identified several limitations in current research. Many studies relied on small, homogenous datasets, which may limit the generalizability of the results. The variability in data acquisition methods and patient demographics also poses challenges for standardizing ML models across different clinical settings. Furthermore, while DL models have shown high performance, their complexity and lack of interpretability can hinder their acceptance and integration into clinical practice.

### Future research suggestions

• **Expand and Diversify Datasets**: Future research should focus on collecting larger and more diverse datasets to improve the generalizability of ML models. This includes incorporating data from different ethnic groups, clinical settings, and geographic locations.

• **Enhance Model Interpretability**: Developing methods to improve the transparency and interpretability of DL models will be crucial for their acceptance in clinical practice. Techniques such as attention mechanisms and XAI can be explored to provide insights into model decision-making processes.

• **Standardize Data Acquisition and Preprocessing**: Establishing standardized protocols for data acquisition and preprocessing can help reduce variability and improve the reliability of ML models. This includes creating guidelines for handling data imbalance and feature selection to ensure consistency across studies.

## Electronic supplementary material

Below is the link to the electronic supplementary material.

Supplementary Material 1

## Data Availability

All data generated or analysed during this study are included in this published article.

## References

[1] Tham Y-C, Li X, Wong TY, Quigley HA, Aung T, Cheng C-Y. Global prevalence of glaucoma and projections of glaucoma burden through 2040: a systematic review and meta-analysis. Ophthalmology. 2014;121:2081–90. 10.1016/j.ophtha.2014.05.013. 24974815 10.1016/j.ophtha.2014.05.013

[2] Allison K, Patel D, Alabi O. Epidemiology of glaucoma: the past, present, and predictions for the future. Cureus. 2020;12. 10.7759/cureus.11686. 10.7759/cureus.11686 PMC7769798 33391921

[3] Tanna AP, Ophthalmology AAo. 2022–2023 Basic and Clinical Science Course, Sect. 10: Glaucoma. American Academy of Ophthalmology; 2022.

[4] Malihi M, Moura Filho ER, Hodge DO, Sit AJ. Long-term trends in glaucoma-related blindness in Olmsted County, Minnesota. *Ophthalmology* 121, 134–141. 10.1016/j.ophtha.2013.09.003 (2014). 10.1016/j.ophtha.2013.09.003 PMC4038428 24823760

[5] Rylander NR, Vold SD. Cost analysis of glaucoma medications. Am J Ophthalmol. 2008;145:106–13. 10.1016/j.ajo.2007.08.041. 18154755 10.1016/j.ajo.2007.08.041

[6] Rouland J-F, Berdeaux G, Lafuma A. The economic burden of glaucoma and ocular hypertension: implications for patient management: a review. Drugs Aging. 2005;22:315–21. 10.2165/00002512-200522040-00004. 15839720 10.2165/00002512-200522040-00004

[7] Gordon MO, et al. The ocular hypertension treatment study: baseline factors that predict the onset of primary Open-Angle Glaucoma. Arch Ophthalmol. 2002;120:714–20. 10.1001/archopht.120.6.714. 12049575 10.1001/archopht.120.6.714

[8] Gedde SJ, et al. Primary Open-Angle Glaucoma preferred practice Pattern^®^. Ophthalmology. 2021;128:P71–150. 10.1016/j.ophtha.2020.10.022. 34933745 10.1016/j.ophtha.2020.10.022

[9] Miglior S, Zeyen T, Pfeiffer N, Cunha-Vaz J, Torri V, Adamsons I. Results of the European Glaucoma prevention study. Ophthalmology. 2005;112:366–75. 10.1016/j.ophtha.2004.11.030. 15745761 10.1016/j.ophtha.2004.11.030

[10] Shaarawy TM, Sherwood MB, Hitchings RA, Crowston JG. Glaucoma. Elsevier Health Sciences; 2014.

[11] Medeiros FA, Lisboa R, Weinreb RN, Liebmann JM, Girkin C, Zangwill LM. Retinal ganglion cell count estimates associated with early development of visual field defects in glaucoma. Ophthalmology. 2013;120:736–44. 10.1016/j.ophtha.2012.09.039. 23246120 10.1016/j.ophtha.2012.09.039 PMC3804164

[12] Wu Y, et al. Measures of disease activity in glaucoma. Biosens Bioelectron. 2022;196:113700. 10.1016/j.bios.2021.113700. 34653715 10.1016/j.bios.2021.113700

[13] Chan TCW, Bala C, Siu A, Wan F, White A. Risk factors for rapid glaucoma disease progression. Am J Ophthalmol. 2017;180:151–7. 10.1016/j.ajo.2017.06.003. 28624324 10.1016/j.ajo.2017.06.003

[14] Comparison of glaucomatous progression. Between untreated patients with normal-tension glaucoma and patients with therapeutically reduced intraocular pressures. Collaborative Normal-Tension Glaucoma study group. Am J Ophthalmol. 1998;126:487–97. 10.1016/s0002-9394(98)00223-2. 9780093 10.1016/s0002-9394(98)00223-2

[15] Deokule S, Weinreb RN. Relationships among systemic blood pressure, intraocular pressure, and open-angle glaucoma. Can J Ophthalmol. 2008;43:302–7. 10.3129/i08-061. 18493272 10.3129/i08-061

[16] Stagg BC, et al. Special commentary: using clinical decision support systems to bring predictive models to the Glaucoma clinic. Ophthalmol Glaucoma. 2021;4:5–9. 10.1016/j.ogla.2020.08.006. 32810611 10.1016/j.ogla.2020.08.006 PMC7854795

[17] Berner ES, La Lande TJ. Clinical decision support systems. Springer; 2007. pp. 3–22.

[18] Zheng C, Johnson TV, Garg A, Boland MV. Artificial intelligence in glaucoma. Curr Opin Ophthalmol. 2019;30:97–103. 10.1097/ICU.0000000000000552. 30562242 10.1097/ICU.0000000000000552

[19] Salazar H, Misra V, Swaminathan SS. Artificial intelligence and complex statistical modeling in glaucoma diagnosis and management. Curr Opin Ophthalmol. 2021;32:105–17. 10.1097/ICU.0000000000000741. 33395111 10.1097/ICU.0000000000000741

[20] Chen JS, et al. Usability and clinician acceptance of a deep Learning-Based clinical decision support tool for predicting glaucomatous visual field progression. J Glaucoma. 2023;32:151–8. 10.1097/IJG.0000000000002163. 36877820 10.1097/IJG.0000000000002163 PMC9996451

[21] Gomes B, Ashley EA. Artificial intelligence in molecular medicine. N Engl J Med. 2023;388:2456–65. 10.1056/NEJMra2204787. 37379136 10.1056/NEJMra2204787

[22] Shehab M, et al. Machine learning in medical applications: A review of state-of-the-art methods. Comput Biol Med. 2022;145:105458. 10.1016/j.compbiomed.2022.105458. 35364311 10.1016/j.compbiomed.2022.105458

[23] Gulshan V, et al. Development and validation of a deep learning algorithm for detection of diabetic retinopathy in retinal fundus photographs. JAMA. 2016;316:2402–10. 10.1001/jama.2016.17216. 27898976 10.1001/jama.2016.17216

[24] Shahriari MH, Sabbaghi H, Asadi F, Hosseini A, Khorrami Z. Artificial intelligence in screening, diagnosis, and classification of diabetic macular edema: A systematic review. Surv Ophthalmol. 2023;68:42–53. 10.1016/j.survophthal.2022.08.004. 35970233 10.1016/j.survophthal.2022.08.004

[25] Niazi S, et al. Keratoconus diagnosis: from fundamentals to artificial intelligence: A systematic narrative review. Diagnostics. 2023;13:2715. 10.3390/diagnostics13162715. 37627975 10.3390/diagnostics13162715 PMC10453081

[26] Stagg BC, et al. Systematic User-centered design of a prototype clinical decision support system for Glaucoma. Ophthalmol Sci. 2023;3. 10.1016/j.xops.2023.100279. 10.1016/j.xops.2023.100279 PMC10033738 36970116

[27] Krishnan S, Amudha J, Tejwani S. Intelligent-based decision support system for diagnosing glaucoma in primary eyecare centers using eye tracker. J Intell Fuzzy Syst. 2021;41:5235–42. 10.3233/JIFS-189846.

[28] Acharya UR, et al. Decision support system for the glaucoma using Gabor transformation. Biomed Signal Process Control. 2015;15:18–26. 10.1016/j.bspc.2014.09.004.

[29] Coan LJ, et al. Automatic detection of glaucoma via fundus imaging and artificial intelligence: A review. Surv Ophthalmol. 2023;68:17–41. 10.1016/j.survophthal.2022.08.005. 35985360 10.1016/j.survophthal.2022.08.005

[30] Zhang L, Tang L, Xia M, Cao G. The application of artificial intelligence in glaucoma diagnosis and prediction. Front Cell Dev Biol. 2023;11:1173094. 10.3389/fcell.2023.1173094. 37215077 10.3389/fcell.2023.1173094 PMC10192631

[31] Chen D, et al. Applications of artificial intelligence and deep learning in Glaucoma. Asia Pac J Ophthalmol (Phila). 2023;12:80–93. 10.1097/apo.0000000000000596. 36706335 10.1097/APO.0000000000000596

[32] Liao WM, Zou BJ, Zhao RC, Chen YQ, He ZY, Zhou MJ. Clinical interpretable deep learning model for Glaucoma diagnosis. Ieee J Biomedical Health Inf. 2020;24:1405–12. 10.1109/jbhi.2019.2949075. 10.1109/JBHI.2019.2949075 31647449

[33] Olivas LG, Alférez GH, Castillo J. Glaucoma detection in Latino population through oct’s RNFL thickness map using transfer learning. Int Ophthalmol. 2021;41:3727–41. 10.1007/s10792-021-01931-w. 34212255 10.1007/s10792-021-01931-w

[34] García G, Colomer A, Naranjo V. Glaucoma detection from Raw SD-OCT volumes: A novel approach focused on Spatial dependencies. Comput Methods Programs Biomed. 2021;200. 10.1016/j.cmpb.2020.105855. 10.1016/j.cmpb.2020.105855 33303289

[35] Raju M, Shanmugam KP, Shyu CR. Application of machine learning predictive models for early detection of Glaucoma using real world data. Appl Sci (Switzerland). 2023;13. 10.3390/app13042445.

[36] Li C, et al. Assessing the external validity of machine learning-based detection of glaucoma. Sci Rep. 2023;13. 10.1038/s41598-023-27783-1. 10.1038/s41598-023-27783-1 PMC9834286 36631567

[37] Oh S, Park Y, Cho KJ, Kim SJ. Explainable machine learning model for glaucoma diagnosis and its interpretation. Diagnostics. 2021;11. 10.3390/diagnostics11030510. 10.3390/diagnostics11030510 PMC8001225 33805685

[38] Page MJ et al. The PRISMA 2020 statement: an updated guideline for reporting systematic reviews. *bmj* 372. 10.1136/bmj.n71 (2021). 10.1136/bmj.n71 PMC8005924 33782057

[39] QUADAS-2: A Revised Tool for the Quality Assessment of Diagnostic Accuracy Studies. Ann Intern Med. 2011;155:529–36. 10.7326/0003-4819-155-8-201110180-00009. 22007046 10.7326/0003-4819-155-8-201110180-00009

[40] Omar Y, ElSheikh MA, Hodhod R, GLAUDIA. A predicative system for glaucoma diagnosis in mass scanning. Health Inf J. 2021;27:14604582211009276. 10.1177/14604582211009276. 10.1177/14604582211009276 33913369

[41] Kaskar OG, Wells-Gray E, Fleischman D, Grace L. Evaluating machine learning classifiers for glaucoma referral decision support in primary care settings. Sci Rep. 2022;12. 10.1038/s41598-022-12270-w. 10.1038/s41598-022-12270-w PMC9122936 35595794

[42] Huang XQ, et al. An objective and Easy-to-Use Glaucoma functional severity staging system based on artificial intelligence. J Glaucoma. 2022;31:626–33. 10.1097/ijg.0000000000002059. 35658070 10.1097/IJG.0000000000002059 PMC9378471

[43] Sharifi M, Khatibi T, Emamian MH, Sadat S, Hashemi H, Fotouhi A. Development of glaucoma predictive model and risk factors assessment based on supervised models. BioData Min. 2021;14. 10.1186/s13040-021-00281-8. 10.1186/s13040-021-00281-8 PMC8611977 34819128

[44] Eswari MS, Balamurali S. in *2021 International Conference on Advance Computing and Innovative Technologies in Engineering, ICACITE 2021.* 447–449.

[45] Lazouni MEA, Feroui A, Mahmoudi S. A new intelligent system for glaucoma disease detection. Int J Comput Aided Eng Technol. 2019;11:613–33. 10.1504/IJCAET.2019.100457.

[46] Wyawahare MV, Patil PM. Machine learning classifiers based on structural ONH measurements for glaucoma diagnosis. Int J BioMed Eng Technol. 2016;21:343–60. 10.1504/IJBET.2016.078338.

[47] Barella KA, Costa VP, Vidotti VG, Silva FR, Dias M, Gomi ES. Glaucoma Diagnostic Accuracy of Machine Learning Classifiers Using Retinal Nerve Fiber Layer and Optic Nerve Data from SD-OCT. *Journal of Ophthalmology* 2013, 10.1155/2013/789129 (2013). 10.1155/2013/789129 PMC3863536 24369495

[48] Kooner KS, et al. Glaucoma diagnosis through the integration of optical coherence tomography/angiography and machine learning diagnostic models. Clin Ophthalmol. 2022;16:2685–97. 10.2147/OPTH.S367722. 36003072 10.2147/OPTH.S367722 PMC9394657

[49] Ibrahim MH, Hacibeyoglu M, Agaoglu A, Ucar F. Glaucoma disease diagnosis with an artificial algae-based deep learning algorithm. Med Biol Eng Comput. 2022;60:785–96. 10.1007/s11517-022-02510-6. 35080695 10.1007/s11517-022-02510-6

[50] Rabiolo A, et al. Combining structural and vascular parameters to discriminate among Glaucoma patients, Glaucoma suspects, and healthy subjects. Translational Vis Sci Technol. 2021;10:20. 10.1167/tvst.10.14.20. 10.1167/tvst.10.14.20 PMC8709930 34928324

[51] Leite D et al. in Procedia Comput Sci 454–60. https://www.sciencedirect.com/science/article/pii/S1877050921022596

[52] Kim SJ, Cho KJ, Oh S. Development of machine learning models for diagnosis of glaucoma. PLoS ONE. 2017;12. 10.1371/journal.pone.0177726. 10.1371/journal.pone.0177726 PMC5441603 28542342

[53] Vidotti VG, et al. Sensitivity and specificity of machine learning classifiers and spectral domain OCT for the diagnosis of glaucoma. Eur J Ophthalmol. 2013;23:61–9. 10.5301/ejo.5000183. 10.5301/ejo.5000183 22729440

[54] Hatanaka Y, Muramatsu C, Sawada A, Hara T, Yamamoto T, Fujita H. in *Proceedings of the Annual International Conference of the IEEE Engineering in Medicine and Biology Society, EMBS.* 5963–5966. 10.1109/EMBC.2012.6347352 23367287

[55] Wu CW, Chen HY, Chen JY, Lee CH. Glaucoma Detection Using Support Vector Machine Method Based on Spectralis OCT. *Diagnostics* 12. 10.3390/diagnostics12020391 (2022). 10.3390/diagnostics12020391 PMC8871188 35204482

[56] Huang XL, et al. A Structure-Related Fine-Grained deep learning system with diversity data for universal Glaucoma visual field grading. Front Med. 2022;9. 10.3389/fmed.2022.832920. 10.3389/fmed.2022.832920 PMC8968343 35372429

[57] Akter N, Fletcher J, Perry S, Simunovic MP, Briggs N, Roy M. Glaucoma diagnosis using multi-feature analysis and a deep learning technique. Sci Rep. 2022;12. 10.1038/s41598-022-12147-y. 10.1038/s41598-022-12147-y PMC9110703 35577876

[58] Lee SD, Lee JH, Choi YG, You HC, Kang JH, Jun CH. Machine learning models based on the dimensionality reduction of standard automated perimetry data for glaucoma diagnosis. Artif Intell Med. 2019;94:110–6. 10.1016/j.artmed.2019.02.006. 30871677 10.1016/j.artmed.2019.02.006

[59] Asaoka R, Murata H, Iwase A, Araie M. Detecting preperimetric glaucoma with standard automated perimetry using a deep learning classifier. Ophthalmology. 2016;123:1974–80. 10.1016/j.ophtha.2016.05.029. 27395766 10.1016/j.ophtha.2016.05.029

[60] Asaoka R, Iwase A, Hirasawa K, Murata H, Araie M. Identifying preperimetric glaucoma in standard automated perimetry visual fields. Investig Ophthalmol Vis Sci. 2014;55:7814–20. 10.1167/iovs.14-15120. 25342615 10.1167/iovs.14-15120

[61] Yoshida T, et al. Discriminating between glaucoma and normal eyes using optical coherence tomography and the ‘random forests’ classifier. PLoS ONE. 2014;9. 10.1371/journal.pone.0106117. 10.1371/journal.pone.0106117 PMC4148397 25167053

[62] Bizios D, Heijl A, Bengtsson B. Integration and fusion of standard automated perimetry and optical coherence tomography data for improved automated glaucoma diagnostics. BMC Ophthalmol. 2011;11. 10.1186/1471-2415-11-20. 10.1186/1471-2415-11-20 PMC3167760 21816080

[63] Huang ML, Chen HY, Huang WC, Tsai YY. Linear discriminant analysis and artificial neural network for glaucoma diagnosis using scanning laser polarimetry-variable cornea compensation measurements in Taiwan Chinese population. Graefe’s Archive Clin Experimental Ophthalmol. 2010;248:435–41. 10.1007/s00417-009-1259-3. 10.1007/s00417-009-1259-3 20012983

[64] Escamez CSF, Giral EM, Martinez SP, Fernandez NT. High interpretable machine learning classifier for early glaucoma diagnosis. Int J Ophthalmol. 2021;14:393–8. 10.18240/ijo.2021.03.10. 33747815 10.18240/ijo.2021.03.10 PMC7930548

[65] Lu SH, Lee KY, Chong JIT, Lam AKC, Lai JSM, Lam DCC. in *Proceedings– 2018 IEEE International Conference on Bioinformatics and Biomedicine, BIBM* 2018. 2539–2543.

[66] Shigueoka LS, et al. Automated algorithms combining structure and function outperform general ophthalmologists in diagnosing glaucoma. PLoS ONE. 2018;13:e0207784. 10.1371/journal.pone.0207784. 30517157 10.1371/journal.pone.0207784 PMC6281287

[67] Yoo TK, Hong S. Artificial neural network approach for differentiating open-angle glaucoma from glaucoma suspect without a visual field test. Investig Ophthalmol Vis Sci. 2015;56:3957–66. 10.1167/iovs.15-16805. 26098462 10.1167/iovs.15-16805

[68] Sugimoto K, Murata H, Hirasawa H, Aihara M, Mayama C, Asaoka R. Cross-sectional study: Does combining optical coherence tomography measurements using the ‘Random Forest’ decision tree classifier improve the prediction of the presence of perimetric deterioration in glaucoma suspects? BMJ Open. 2013;3. 10.1136/bmjopen-2013-003114. 10.1136/bmjopen-2013-003114 PMC3796272 24103806

[69] Boland MV, Quigley HA. Evaluation of a combined index of optic nerve structure and function for glaucoma diagnosis. BMC Ophthalmol. 2011;11:1–12. 10.1186/1471-2415-11-6. 21314957 10.1186/1471-2415-11-6 PMC3047293

[70] Racette L, et al. Combining functional and structural tests improves the diagnostic accuracy of relevance vector machine classifiers. J Glaucoma. 2010;19:167–75. 10.1097/IJG.0b013e3181a98b85. 19528827 10.1097/IJG.0b013e3181a98b85 PMC2891254

[71] Bizios D, Heijl A, Hougaard JL, Bengtsson B. Machine learning classifiers for glaucoma diagnosis based on classification of retinal nerve fibre layer thickness parameters measured by stratus OCT. Acta Ophthalmol. 2010;88:44–52. 10.1111/j.1755-3768.2009.01784.x. 20064122 10.1111/j.1755-3768.2009.01784.x

[72] Gardiner SK, Johnson CA, Cioffi GA. Evaluation of the structure-function relationship in glaucoma. Investig Ophthalmol Vis Sci. 2005;46:3712–7. 10.1167/iovs.05-0266. 16186353 10.1167/iovs.05-0266

[73] Sung KR, Kim JS, Wollstein G, Folio L, Kook MS, Schuman JS. Imaging of the retinal nerve fibre layer with spectral domain optical coherence tomography for glaucoma diagnosis. Br J Ophthalmol. 2011;95:909–14. 10.1136/bjo.2010.186924. 21030413 10.1136/bjo.2010.186924 PMC3421150

[74] Lisboa R, Leite MT, Zangwill LM, Tafreshi A, Weinreb RN, Medeiros FA. Diagnosing preperimetric glaucoma with spectral domain optical coherence tomography. Ophthalmology. 2012;119:2261–9. 10.1016/j.ophtha.2012.06.009. 22883689 10.1016/j.ophtha.2012.06.009 PMC3787835

[75] Doughty MJ, Zaman ML. Human corneal thickness and its impact on intraocular pressure measures: a review and meta-analysis approach. Surv Ophthalmol. 2000;44:367–408. 10.1016/s0039-6257(00)00110-7. 10734239 10.1016/s0039-6257(00)00110-7

[76] Shoukat A, Akbar S, Hassan SA, Iqbal S, Mehmood A, Ilyas QM. Automatic diagnosis of Glaucoma from retinal images using deep learning approach. Diagnostics. 2023;13:1738. 10.3390/diagnostics13101738. 37238222 10.3390/diagnostics13101738 PMC10217711

[77] Guo J-M, et al. A study of the interpretability of fundus analysis with deep Learning-Based approaches for Glaucoma assessment. Electronics. 2013;12. 10.3390/electronics12092013. (2023).

[78] Liu B, Li S-q, He R-l, Zhao. Y.-g. in *2023 IEEE 12th Data Driven Control and Learning Systems Conference (DDCLS).* 1492–1497 (IEEE).

[79] Hastie T, Tibshirani R, Friedman JH, Friedman JH. The elements of statistical learning: data mining, inference, and prediction. Volume 2. Springer; 2009.

[80] Domingos P. A few useful things to know about machine learning. Commun ACM. 2012;55:78–87. 10.1145/2347736.2347755.

[81] Raschka S, Mirjalili V. Python machine learning: machine learning and deep learning with python, scikit-learn, and tensorflow 2. Packt Publishing Ltd; 2019.

[82] Kaur H, Pannu H, Malhi AA, Systematic. Review on imbalanced data challenges in machine learning. ACM Comput Surv (CSUR). 2019;52:1–36. 10.1145/3343440.

[83] Mohindru G, Mondal K, Banka H. Different hybrid machine intelligence techniques for handling IoT-based imbalanced data. CAAI Trans Intell Technol. 2021;6:405–16. 10.1049/CIT2.12032.

[84] Lemaître G, Nogueira F, Aridas C. Imbalanced-learn: A Python toolbox to tackle the curse of imbalanced datasets in machine learning. J Mach Learn Res. 2016;18:17–17.

[85] Safran C, et al. Toward a National framework for the secondary use of health data: an American medical informatics association white paper. J Am Med Inform Assoc. 2007;14:1–9. 10.1197/jamia.M2273. 17077452 10.1197/jamia.M2273 PMC2329823

[86] Bowd C, Goldbaum MH. Machine learning classifiers in glaucoma. Optom Vis Sci. 2008;85:396–405. 10.1097/OPX.0b013e3181783ab6. 18521021 10.1097/OPX.0b013e3181783ab6

[87] Medeiros FA, Zangwill LM, Girkin CA, Liebmann JM, Weinreb RN. Combining structural and functional measurements to improve estimates of rates of glaucomatous progression. Am J Ophthalmol. 2012;153:1197–205. 10.1016/j.ajo.2011.11.015. e1191, doi. 22317914 10.1016/j.ajo.2011.11.015 PMC3804258

[88] Racette L, et al. Combining functional and structural tests improves the diagnostic accuracy of relevance vector machine classifiers. J Glaucoma. 2010;19:167. 10.1097/IJG.0b013e3181a98b85. 19528827 10.1097/IJG.0b013e3181a98b85 PMC2891254

[89] Weinreb RN, Aung T, Medeiros FA. The pathophysiology and treatment of glaucoma: a review. JAMA. 2014;311:1901–11. 10.1001/jama.2014.3192. 24825645 10.1001/jama.2014.3192 PMC4523637

[90] De Moraes CG, Cioffi GA, Weinreb RN, Liebmann JM. Perspective: new recommendations for the treatment of systemic hypertension and their potential implications for glaucoma management. J Glaucoma. 2018;27:567. 10.1097/IJG.0000000000000981. 29750712 10.1097/IJG.0000000000000981 PMC6028320

