# Supplementary material for: Applications of machine learning in glaucoma diagnosis based on tabular data: a systematic review
Source: BMC Biomed Eng. 2025 Aug 1;7:9. doi: 10.1186/s42490-025-00095-3 (PMC12315471; doi:10.1186/s42490-025-00095-3)
Supplement: Supplementary file 1 — Supplementary Material 1 [file 42490_2025_95_MOESM1_ESM.docx]

Sup Table 1. QUADAS-2 Checklist for all selected studies.

| **Study** | **Risk of bias** | | | | **Applicability Concerns** | | | |  |
| --- | --- | --- | --- | --- | --- | --- | --- | --- | --- |
|  | **Patient selection** | **Index test** | **Reference standard** | **Flow and timing** | | **Patient selection** | **Index test** | **Reference standard** | |
| **Li, et al. 2023 ^54^** | low | low | low | low | | low | low | low | |
| **Raju, et al. 2023 ^75^** | low | low | low | low | | low | low | low | |
| **Ibrahim, et al. 2022 ^44^** | unclear | low | low | unclear | | low | low | low | |
| **Akter et al, 2022 ^2^** | low | low | low | low | | low | low | low | |
| **Kooner, et al. 2022 ^49^** | low | low | low | low | | low | low | low | |
| **Wu, et al. 2022 ^97^** | low | unclear | low | unclear | | low | low | low | |
| **Kaskar, et al. 2022 ^46^** | low | low | low | low | | low | low | low | |
| **Huang, et al. 2022 ^43^** | low | low | unclear | unclear | | low | low | unclear | |
| **Huang, Jin et al. 2022 ^42^** | low | low | unclear | unclear | | low | low | low | |
| **Leite, et al. 2021 ^53^** | low | low | unclear | low | | low | low | low | |
| **Escamez, et al. 2021 ^24^** | low | low | low | low | | low | low | low | |
| **Omar, et al. 2021 ^70^** | high | unclear | low | low | | unclear | low | low | |
| **Oh, et al. 2021 ^68^** | low | low | low | low | | low | low | low | |
| **Sharifi, et al. 2021 ^83^** | low | low | low | low | | low | low | low | |
| **Rabiolo, et al. 2021 ^72^** | low | low | unclear | low | | low | low | low | |
| **Eswari, et al. 2021 ^25^** | low | low | low | low | | low | low | low | |
| **Lee, et al. 2021 ^52^** | low | low | low | low | | low | low | low | |
| **Lu, et al. 2021 ^58^** | low | low | low | low | | low | low | low | |
| **Lazouni, et al. 2019 ^51^** | low | low | low | low | | low | low | low | |
| **Shigueoka, et al. 2018 ^85^** | low | low | unclear | low | | low | low | low | |
| **Kim, et al. 2017 ^48^** | low | low | unclear | low | | low | low | low | |
| **Wyawahare, 2016 ^100^** | low | low | low | low | | low | low | low | |
| **Asaoka, et al. 2016 ^5^** | low | low | low | low | | low | low | low | |
| **Yoo, et al. 2015 ^101^** | low | low | unclear | low | | low | low | low | |
| **Asaoka, et al. 2014 ^4^** | low | low | low | low | | low | low | low | |
| **Yoshida, et al. 2014 ^102^** | low | low | unclear | low | | low | low | low | |
| **Vidotti, et al. 2013 ^95^** | low | low | low | low | | low | low | unclear | |
| **Barella, et al. 2013 ^6^** | low | low | unclear | low | | low | low | low | |
| **Sugimoto, et al. 2013 ^89^** | low | low | low | low | | low | low | low | |
| **Hatanaka, et al. 2012 ^38^** | low | low | low | low | | low | low | low | |
| **Bizios, et al. 2011 ^8^** | low | low | low | low | | low | low | low | |
| **Boland, et al. 2011 ^10^** | low | unclear | low | low | | low | low | low | |
| **Racette, et al. 2010 ^74^** | low | low | low | low | | low | low | low | |
| **Bizios, et al. 2010 ^9^** | low | low | low | low | | low | low | low | |
| **Huang, et al. 2010 ^41^** | low | unclear | low | low | | low | low | low | |

|  |  |  |  |  |  |  |
| --- | --- | --- | --- | --- | --- | --- |
